# Toward the Synthesis and Improved Biopotential of an *N*-methylated Analog of a Proline-Rich Cyclic Tetrapeptide from Marine Bacteria

**DOI:** 10.3390/md16090305

**Published:** 2018-08-30

**Authors:** Rajiv Dahiya, Suresh Kumar, Sukhbir Lal Khokra, Sheeba Varghese Gupta, Vijaykumar B. Sutariya, Deepak Bhatia, Ajay Sharma, Shamjeet Singh, Sandeep Maharaj

**Affiliations:** 1 Laboratory of Peptide Research and Development, School of Pharmacy, Faculty of Medical Sciences, The University of the West Indies, Saint Augustine, Trinidad and Tobago; Shamjeet.Singh@sta.uwi.edu (S.S.); Sandeep.Maharaj@sta.uwi.edu (S.M.); 2 Institute of Pharmaceutical Sciences, Kurukshetra University, Kurukshetra 136119, Haryana, India; slkhokra@kuk.ac.in; 3 Department of Pharmaceutical Sciences, USF College of Pharmacy, University of South Florida, Tampa, FL 33612-4749, USA; svarghes@health.usf.edu (S.V.G.); vsutariy@health.usf.edu (V.B.S.); 4 Department of Pharmacogenomics, ICPH Fairfax Bernard J. Dunn School of Pharmacy, Shenandoah University, Fairfax, VA 22031, USA; dbhatia@su.edu; 5 Department of Pharmacognosy, Amity Institute of Pharmacy, Amity University, Gwalior 474020, Madhya Pradesh, India; asharma5@gwa.amity.edu

**Keywords:** *N*-methylation, tetracyclopeptide, *Pseudomonas* sp., macrocyclization, marine sponge, *Pseudoalteromonas* sp., solution-phase peptide synthesis, pharmacological activity

## Abstract

An *N*-methylated analog of a marine bacteria-derived natural proline-rich tetracyclopeptide was synthesized by coupling the deprotected dipeptide fragments Boc-l-prolyl-l-*N*-methylleucine-OH and l-prolyl-l-*N*-methylphenylalanine-OMe. A coupling reaction was accomplished utilizing *N*,*N*′-Dicyclohexylcarbodidimde (DCC) and 1-Ethyl-3-(3-dimethylaminopropyl) carbodiimide (EDC·HCl) as coupling agents and Triethylamine (TEA) or *N*-methylmorpholine (NMM) as the base in the presence of the racemization suppressing agent. This was followed by the cyclization of the linear tetrapeptide fragment under alkaline conditions. The structure of the synthesized cyclooligopeptide was confirmed using quantitative elemental analysis, FTIR (Fourier-transform infrared spectroscopy), ^1^H NMR (Nuclear magnetic resonance spectroscopy), ^13^C NMR, and mass spectrometry. From the bioactivity results, it was clear that the newly synthesized proline-rich tetracyclopeptide exhibited better anthelmintic potential against *Megascoplex konkanensis*, *Pontoscotex corethruses*, and *Eudrilus eugeniae* at a concentration of 2 mg/mL as well as improved antifungal activity against pathogenic dermatophytes *Trichophyton mentagrophytes* and *Microsporum audouinii* at a concentration of 6 μg/mL, as compared to non-methylated tetracyclopeptide. Moreover, *N*-methylated tetracyclopeptide displayed significant activity against pathogenic *Candida albicans*.

## 1. Introduction

The marine environment is distinguished by unique groups of organisms which are a source of extremely interesting structures with a wide array of biological activities [1]. The enormous biodiversity of marine habitats is mirrored by the molecular diversity of secondary metabolites found to be associated with marine sponges, bacteria, fungi, mollusks, etc. Among these metabolites, cyclopeptides have emerged as a unique group of bioactive analogs with interesting pharmacological and biochemical properties including cytotoxicity [2,3,4], insecticidal activity [5], antimicrobial activity [6,7], antitubercular activity [8], anti-inflammatory activity [9], Human Immunodeficiency Virus (HIV)-inhibitory activity [10], chymotrypsin-inhibitory activity [11], antimalarial activity [12], etc. Among cyclooligopeptides, *N*-methylated peptides have attracted the attention of researchers and scientists in terms of their unique structures and diverse pharmacological activities [13,14]. Evolutionarily speaking, nature has employed the *N*-methylation of peptides as an ingenious technique to modulate biological function [15]. The *N*-methylation of amino acids and their derivatives can be carried out with dimethyl sulfate in the presence of sodium hydride and a catalytic amount of water [16], by utilizing 2-chlorotrityl chloride resin/nosyl group and diazomethane [17], by the reductive amination, and via other novel methods [18]. In recent years, through the advancement in synthetic approaches, the potential of *N*-methylation has begun to be revealed in terms of modulating the biological activity, selectivity, and pharmacokinetic properties of peptides, but also in delivering novel drugs [19,20]. Many studies have shown the *N*-methylation of the cyclic peptides to offer a number of advantages, including the improvement of the therapeutic efficacy of peptides by fine-tuning their selectivity for a receptor, enhancing the hydrophobicity by reducing the number of hydrogen-bond donors and by preventing the formation of intermolecular and intramolecular hydrogen bonds, which in turn improves the oral bioavailability [21].

Keeping in view the utilization of *N*-methylation in modulating the biological properties of peptides and, further, in continuation of the efforts of our research group in the synthesis of bioactive cyclic peptides [14,22,23,24,25], the present study was directed toward the first total synthesis of an *N*-methylated analog of a natural tetracyclopeptide. The non-methylated natural peptide was previously isolated from the marine bacteria *Pseudomonas* sp. and *Pseudoalteromonas* sp., associated with the seaweed *Diginea* sp. and the sponge *Halisarca ectofibrosa* [26]. Further, the newly synthesized *N*-methylated peptide was subjected to antibacterial, antifungal, antidermatophytic, and anthelmintic screening and its bioproperties were compared with those of the non-methylated analog. 

## 2. Results

### 2.1. Chemistry

The solution-phase technique of peptide synthesis was employed to prepare an *N*-methylated analog of a natural tetracyclopeptide. In the present study, *p*-nitrophenol (*pnp*) was used for the esterification of a linear tetrapeptide chain and compared with pentafluorophenol (*pfp*) during the synthesis of tetracyclopeptide **4**, affording it in a 74–89% yield, utilizing *N*-methylmorpholine and pyridine as bases. There is a literature report indicating the synthesis of non-methylated natural cyclic tetrapeptide employing the solution-phase technique [27], an *N*-methylated analog of which was selected for synthesis in the present investigation. In this study, the peptide units were prepared by the Bodanszky and Bodanszky method with certain modifications [28]. Prior to coupling, the amino groups of l-amino acids were protected using Di-*tert*-butyl dicarbonate (Boc_2_O) and the deprotection of amino groups was achieved using trifluoroacetic acid (TFA). Similarly, the carboxyl groups of l-amino acids were protected by an esterification reaction with Methanol in the presence of Chlorotrimethylsilane and the deprotection of carboxyl groups was realized by alkaline hydrolysis with lithium hydroxide (LiOH). Racemization was avoided by the utilization of 1-Hydroxybenzotriazole (HOBt) in all coupling reactions.

**Figure 1:**
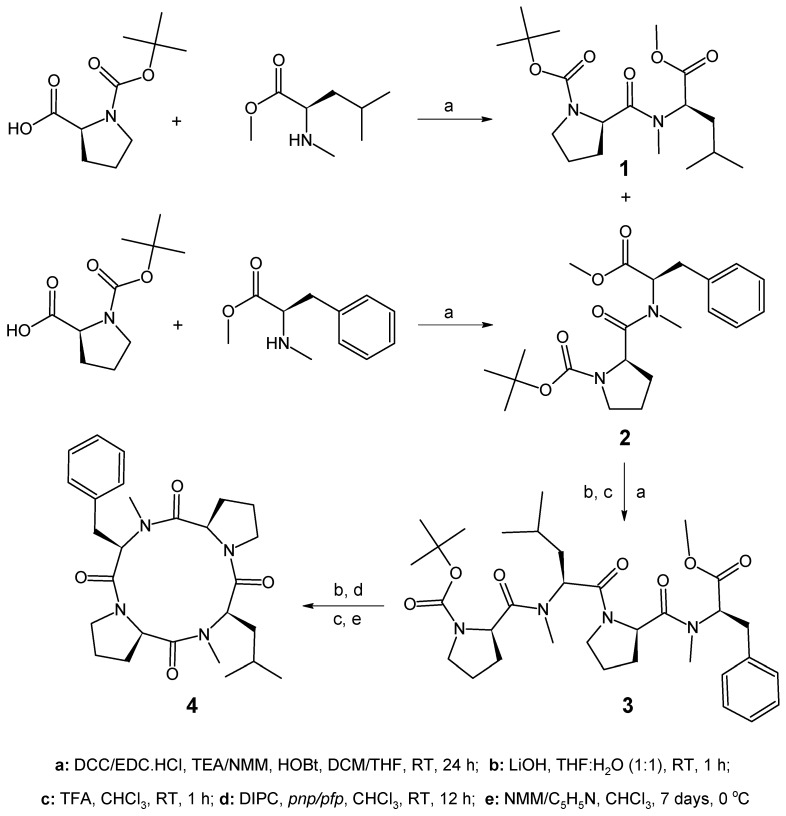
Synthetic route for *N*-methylated proline-rich tetracyclopeptide 4. **1**. Boc-l-Pro-l-*N*-(Me)Leu-OMe; **2**. Boc-l-Pro-l-*N*-(Me)Phe-OMe; **3**. Boc-l-Pro-l-*N*-(Me)Leu-l-Pro-l-*N*-(Me)Phe-OMe; and **4**. cyclo(L-Pro-l-*N*-(Me)Leu-l-Pro-l-*N*-(Me)Phe).

In order to carry out the synthesis of *N*-methylated tetracyclopeptide, Boc-amino acid viz. Boc-l-Pro-OH was coupled with the corresponding *N*-methylated amino acid methyl esters such as l-*N*-(Me)Leu-OMe and l-*N*-(Me)Phe-OMe. The required amino acid methyl ester hydrochloride, such as l-Leu-OMe·HCl, l-Phe-OMe·HCl, and Boc-protected amino acid viz. Boc-l-Pro-OH, were prepared according to previously reported procedures in the literature [29]. The free NH_2_ groups of l-Leu-OMe·HCl and l-Phe-OMe·HCl were protected by the introduction of Boc-groups producing Boc-l-Leu-OMe and Boc-l-Phe-OMe, respectively. The *N*-methylation of Boc-protected leucine and phenylalanine methyl ester was achieved by treatment with methyl iodide and sodium hydride [30], producing Boc-l-*N*-(Me)Leu-OMe and Boc-l-*N*-(Me)Phe-OMe. The Boc-groups of the resulting units were removed using TFA producing l-*N*-(Me)Leu-OMe and l-*N*-(Me)Phe-OMe, respectively. The required dipeptide units Boc-l-Pro-l-*N*-(Me)Leu-OMe (**1**) and Boc-l-Pro-l-*N*-(Me)Phe-OMe (**2**) were prepared by the coupling of *N*-methylated leucine and phenylalanine methyl esters with Boc-l-Pro-OH, employing DCC/EDC·HCl as the coupling agent and TEA as the base. The ester group of the dipeptide unit Boc-l-Pro-l-*N*-(Me)Leu-OMe was removed by alkaline hydrolysis with LiOH and the deprotected peptide was coupled with another dipeptide unit Boc-l-Pro-l-*N*-(Me)Phe-OMe after deprotection at the amino terminal, producing the linear tetrapeptide unit Boc-l-Pro-l-*N*-(Me)Leu-l-Pro-l-*N*-(Me)Phe-OMe (**3**). The methyl ester group of the linear peptide fragment was replaced by a *p*-nitrophenyl (*pnp*)/pentafluorophenyl (*pfp*) ester group. The Boc-group of the resulting compound was removed using TFA and the deprotected linear fragment was then cyclized by maintaining the entire content at 0 °C for 7 days in the presence of catalytic amounts of NMM or pyridine, producing cyclo(l-Pro-l-*N*-(Me)Leu-l-Pro-l-*N*-(Me)Phe) (**4**). The structure of the newly synthesized tetracyclopeptide as well as that of the intermediate di- and tetrapeptides were confirmed by FT-IR, ^1^H/^13^C NMR spectroscopy, and elemental analysis. In addition, mass spectrum was recorded for the *N*-methylated cyclic product **4**. The synthetic pathway for the newly synthesized tetracyclopeptide is given in Figure 1. 

### 2.2. Pharmacological Activity

The anthelmintic screening of linear and cyclic tetracyclopeptides (**3**, **4**) was performed against three earthworm species (*Megascoplex konkanensis*, *Pontoscotex corethruses*, and *Eudrilus eugeniae*) at a concentration of 2 mg/mL using the modified Garg’s method [31]. The bioactivity results are presented in Table 1. Further, antibacterial and antifungal evaluations of peptides **3**, **4** were conducted against four Gram-positive and Gram-negative bacteria—*Bacillus subtilis* and *Staphylococcus aureus*, *Pseudomonas aeruginosa* and *Klebsiella pneumoniae*, respectively; dermatophytes *Microsporum audouinii* and *Trichophyton mentagrophytes*; and diamorphic fungi *Candida albicans* and *Aspergillus niger* using the modified Kirby-Bauer disk diffusion method [32]. The antimicrobial activity results are compiled in Table 2. 

## 3. Discussion

**Figure 2:**
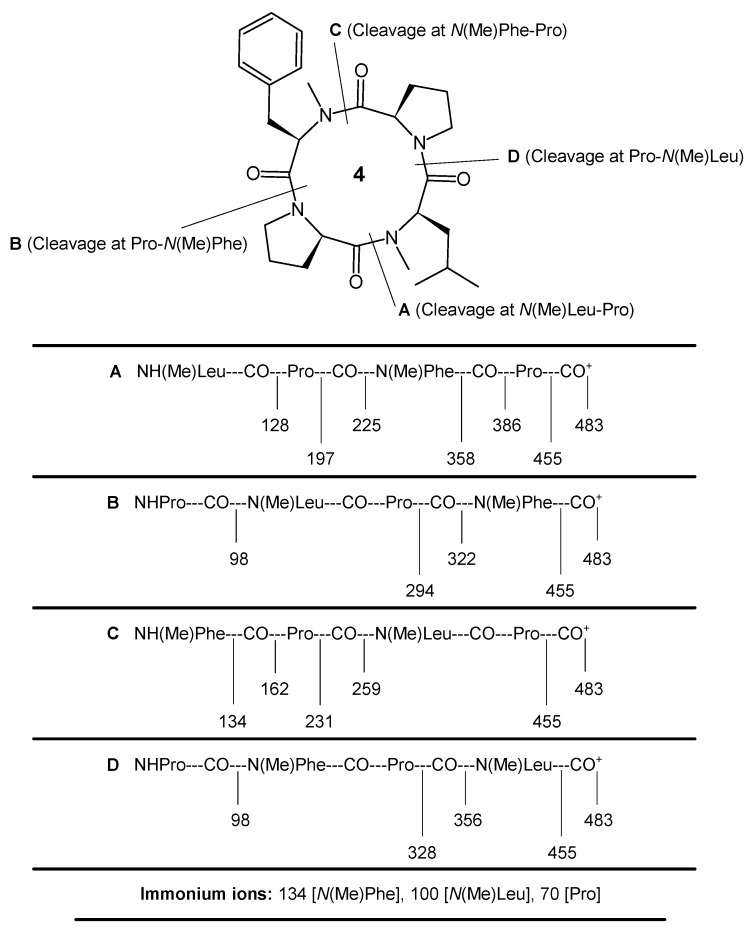
Fragmentation pattern for *N*-methylated proline-rich tetracyclopeptide **4** at diverse amide bond levels.

The synthesis of an *N*-methylated cyclooligopeptide (**4**) was accomplished with an 89% yield, and NMM proved to be an effective base for the cyclization of the linear tetrapeptide segment in comparison to TEA and pyridine. Cyclization was supported by the disappearance of absorption bands at 1742, 1273 and 1395, 1373 cm^−1^ due to C=O_str_, C–O_str_, ester, and C–H_def_, *tert*-butyl groups in the IR spectra of compound **4**. The formation of the cyclopeptide was further confirmed by the disappearance of singlets at 3.54 and 1.52 ppm, corresponding to three protons of the methyl ester group and nine protons of the *tert*-butyl group of Boc in the ^1^H NMR spectrum, in addition to the disappearance of the singlets at 155.4, 80.4 and 53.0, 29.6 ppm, corresponding to carbon atoms of *tert*-butyl and ester groups in the ^13^C NMR spectrum of compound **4**. Furthermore, the ^1^H NMR and ^13^C NMR spectra of the synthesized *N*-methylated tetracyclopeptide showed characteristic peaks confirming the presence of all 38 protons and 27 carbon atoms. The pseudomolecular ion peak (M + 1)^+^ appearing at *m*/*z* = 483 corresponded to the molecular formula C_27_H_38_N_4_O_4_ in the mass spectrum of **4**, along with other fragment ion peaks resulting from the cleavage at ‘*N*(Me)Leu-Pro’, ‘Pro-*N*(Me)Phe’, ‘*N*(Me)Phe-Pro’, and ‘Pro-*N*(Me)Leu’ amide bonds (Figure 2). In addition, the presence of the immonium ion peaks at *m/z* = 134 [*N*(Me)Phe], 100 [*N*(Me)Leu], and 70 [Pro] further confirmed all of the amino acid moieties in the cyclopeptide structure. Furthermore, the elemental analysis of tetracyclopeptide **4** afforded values with a strict tolerance of ±0.03, in accordance with the molecular composition. 

In the present study, we observed that the proline-rich tetracyclopeptide **4** exhibits better anthelmintic activity than the standard drug Mebendazole at a concentration of 2 mg/mL against all three of the tested earthworm species. Comparison of antimicrobial activity data suggested that *N*-methylated tetracyclopeptide **4** possesses potent bioactivity against dermatophytes *M. audouinii*, *T. mentagrophytes* and pathogenic fungi *C. albicans* with minimum inhibitory concentration (MIC) values of 6 μg/mL when compared to the reference drug Griseofulvin. Moreover, moderate activity was seen against Gram-negative bacteria *P. aeruginosa* and *K. pneumonia* for the newly synthesized *N*-methylated tetracyclopeptide, in comparison to the standard drug Gatifloxacin. However, compound **4** displayed little activity against Gram-positive bacteria *S. aureus* but no significant activity against *B. subtitis* nor *A. niger*. In addition, the analysis of the pharmacological activity data revealed that proline-rich *N*-methylated tetracyclopeptide **4** displayed higher bioactivity against pathogenic microbes and earthworms than its linear form, **3**, which is due to the fact that the cyclization of the peptides reduces the degree of freedom for each constituent within the ring and thus substantially leads to the reduced flexibility, as well as increased potency and selectivity of the cyclic peptide [33]. Further, comparison of biological activity data of compound **4** with its non-*N*-methylated analog **IV**, previously synthesized by our research group [27], suggested that proline-rich *N*-methylated tetracyclopeptide **4** displayed improved pharmacological activity against pathogenic dermatophytes, earthworms, and Gram-negative bacteria than its non-*N*-methylated analog **IV**, which is due to the fact that *N*-methylation is the simplest chemical modification occurring in peptides and proteins to improve the potency. In particular, better improvement was observed in the antifungal activity of compound **4** against pathogenic *C. albicans* in comparison to its non-*N*-methylated analog **IV**.

Detailed investigation of the structures and biological potential of naturally occurring cyclooligopeptides suggested that there are various natural cyclic peptides in the literature rich in proline units which have shown biological potential [4]. In most instances, the arrangement of two proline units was found to be alternate to each other, as was also observed in the cyclopeptide chosen for the present study. Besides this, the same type of arrangement of two proline units has been found in the natural/synthetic cyclopeptide ‘Hymenamide E’, which has already shown potent anthelmintic and antifungal effects [29]. This arrangement of two proline units in a cyclic structure in an alternate pattern appears to be responsible for the biological potential of the tetracyclopeptide.

The possible mechanism of action for antimicrobial activity of *N*-methylated proline-rich tetracyclopeptides may involve the active transport inside the bacterial cell where peptides can bind and inactivate the specific targets such as the bacterial ribosome and inhibit protein synthesis, similar to other proline-rich antimicrobial peptides [34]. Antifungal activity may be attributed to the inhibition of chitin synthesis, which is a cell wall component essential to the maintenance of the structural integrity of the fungus; the inhibition of 1–3 β glucan synthase, a multiunit membrane-integrated enzyme which is critical to cell wall integrity; and the ability to traverse the energized membrane and interact with an intracellular target or incite the intracellular induction of reactive oxygen species (ROS), which are toxic to the fungi, as reported for the established anti-fungal peptides [35].

Dermatophytes such as *Microsporum* and *Trichophyton* are the prevailing causes of fungal infection of the skin, hair, and nails due to their ability to utilize keratin. They are also responsible for athlete’s foot. In temperate regions, foot ringworm (athlete’s foot) accounts for 75% of all tinea diseases. These dermatophytes grow in the non-living tissues of hair, nails, and skin, in the region above the layers where keratin is deposited, and cause a complex of diseases known clinically as tinea (ringworm) in humans and other vertebrates [36,37]. The zoophilic fungi, such as *M. audouinii*, more commonly affect children and tend to evoke a more acute inflammatory response than do the anthropophilic fungi. Besides this, *T. mentagrophytes* is among the most common dermatophytes responsible for infections [38]. *Candida albicans* and other *Candida* species can cause cutaneous infections at many sites on the body, especially those that are moist, such as folds of flesh and armpits. *Candida* can cause a different and serious disease if the cells enter and spread within the body [37]. Further, *K. pneumoniae* is an opportunistic pathogen that can cause severe hospital-acquired infections such as septicaemia, pneumonia, urinary tract infection, and soft tissue infection. It is frequently found in the flora of the mouth, skin, and intestines [39]. Another Gram-negative bacterium, *P. aeruginosa*, has emerged as an important pathogen which causes between 10% and 20% of infections in most hospitals. Pseudomonas infection is especially prevalent among patients with burn wounds, cystic fibrosis, acute leukemia, organ transplants, and intravenous-drug addiction [40]. 

As per the literature, and despite its apparent simplicity, *Caenorhabditis elegans* has developed into an important model for biomedical research, particularly in the functional characterization of novel drug targets. Models of the nematode worm *C. elegans* can be used to advance the understanding of the molecular mechanisms of drug action and disease pathogenesis. Several features of *C. elegans*—as it is easy to culture; undergoes rapid reproduction with a short generation time, enabling the large-scale production of animals; has a small size; is transparent, which enables the use of fluorescent markers to study biological processes in vivo; and exhibits cellular complexity—make it a powerful tool for the pharmaceutical industry [41]. Using mutant strains, RNA interference (RNAi), and passive optical techniques, *C. elegans* can be used as an early model to evaluate mechanisms and pathways that formerly would have required more expensive and time-consuming methods. Further, spectroscopic techniques for *C. elegans* are also valuable preliminary tools for finding novel developmental disruptors, anthelminthic agents, neurotransmitter agonists/antagonists, and proteasome regulators [42].

## 4. Materials and Methods 

The melting point was determined by the open capillary method and is uncorrected. The FTIR spectra for all of the synthesized compounds were recorded using an FTIR-8400S Fourier transform spectrophotometer (Shimadzu, Kyoto, Japan). The ^1^H NMR and ^13^C NMR spectra of the tetracyclopeptide and intermediate linear peptide units were recorded on a Bruker AC 300 spectrometer at 300 MHz (Brucker, IL, USA). The mass spectra of *N*-methylated tetracyclopeptide were recorded on a JMS-DX 303 spectrometer (Jeol, Tokyo, Japan). The elemental analysis was performed on a Vario EL III elemental analyzer (Elementar Vario EL III, Hanau, Germany). The optical rotation was measured on an Optics Technology automatic polarimeter (Optics Technology, Delhi, India). The purity of the synthesized tetracyclopeptide and the intermediate linear peptide units was checked by Thin Layer Chromatography (TLC) on precoated silica gel G plates (Kieselgel 0.25 mm, 60G F_254_, Merck, Germany). 

### 4.1. Procedure for the Preparation of N-Methylated Dipeptide Units (***1***, ***2***)

The *N*-methylated-l-amino acid methyl ester (l-*N*-(Me)Leu-OMe (1.59 g, 0.01 mol)) was dissolved in Dichloromethane (DCM, 30 mL). To this, TEA (2.8 mL, 0.021 mol) was added at 0 °C and the reaction mixture was stirred for 15 min. Boc-l-Pro-OH (2.15 g, 0.01 mol) was dissolved in DCM (30 mL) followed by the addition of DCC (2.12 g, 0.01 mol) and HOBt (1.34 g, 0.01 mol). The resulting mixture was added to the above solution with constant shaking and stirring was continued for 24 h. This reaction was repeated using EDC·HCl (1.92 g, 0.01 mol) as the coupling agent instead of DCC. The reaction mixture was filtered and the residue was washed with DCM (25 mL) and added to the filtrate. The filtrate was washed with 5% NaHCO_3_ and saturated NaCl solutions. The organic layer was dried over anhydrous Na_2_SO_4_, filtered, and evaporated in vacuum. The crude product was recrystallized from a mixture of chloroform and petroleum ether (Boiling point (b.p.) 40–60 °C) followed by cooling at 0 °C to obtain the title compound Boc-l-Pro-l-*N*-(Me)Leu-OMe (**1**). Similarly, the *N*-methylated dipeptide unit Boc-l-Pro-l-*N*-(Me)Phe-OMe (**2**) was prepared by coupling l-*N*-(Me)Phe-OMe (1.93 g, 0.01 mol) and Boc-l-Pro-OH (2.15 g, 0.01 mol) under similar conditions.

#### 4.1.1. *tert*-Butyloxycarbonyl-l-Prolyl-l-*N*-Methyl-l-Leucine Methyl Ester (**1**)

Semisolid mass; yield 79%; [*α*]_D_ = −32.1° (*c* = 0.25, MeOH); R*_f_* = 0.62 (CHCl_3_·MeOH—8:2); IR (CHCl_3_): *v* = 2995–2988 (C–Hstr), 2966, 2927 (C–Hstr), 2796 (C–Hstr *N*-Me), 1744 (C=O_str_), 1677, 1638 (C=O_str_), 1398, 1373 (C–Hdef), 1388, 1367 (C–Hdef), 1269 (C–Ostr) cm^−1^; ^1^H NMR (CDCl_3_): *δ* = 4.52 (t, *J* = 6.1 Hz,1H, H-*α*, Leu), 4.10 (t, *J* = 7.15 Hz, 1H, H-α, Pro), 3.65 (s, 3H, *O*CH_3_), 3.25 (t, *J* = 7.2 Hz, 2H, H-*δ*, Pro), 2.99 (s, 3H, *N*CH_3_), 2.57 (q, *J* = 5.65 Hz, 2H, H-*β*, Pro), 1.96–1.88 (m, 2H, H-*γ*, Pro), 1.49 (s, 9H, *tert*-Butyl), 1.45–1.34 (m, 3H, H-*β* and H-*γ*, Leu), 0.94 (d, 6H, *J* = 6.35 Hz, H-*δ*, Leu); ^13^C NMR (CDCl_3_): *δ* = 176.4 (C=O, Pro), 170.3 (C=O, Leu), 159.8 (C=O, Boc), 79.4 (C-*α*, *tert*-Butyl), 60.7 (C-*α*, Pro), 55.9 (C-*α*, Leu), 54.2 (*O*CH_3_), 46.7 (C-*δ*, Pro), 40.7 (C-*β*, Leu), 35.3 (*N*CH_3_), 30.4 (C-*β*, Pro), 29.1 (3C, C-*β*, *tert*-Butyl), 26.6, 24.1 (2C, C-*γ*, Leu and Pro), 21.8 (2C, C-*δ*, Leu); C_18_H_32_N_2_O_5_ (356): calcd. C 60.65, H 9.05, N 7.86; found C 60.66, H 9.02, N 7.88. 

#### 4.1.2. *tert*-Butyloxycarbonyl-l-Prolyl-l-*N*-Methyl-Phenylalanine Methyl Ester (**2**)

Semisolid mass; yield 81%; [*α*]_D_ = +44.9° (*c* = 0.25, MeOH); R*_f_* = 0.48 (CHCl_3_·MeOH—8:2); IR (CHCl_3_): *v* = 2996-2987 (C–Hstr), 2965, 2929, 2877 (C–Hstr), 2802 (C–Hstr *N*-Me), 1741 (C=O_str_), 1672, 1636 (C=Ostr), 1544, 1415 (C=C), 1389, 1377 (C–Hdef), 1273 (C–Ostr), 725, 686 (C–Hdef) cm^−1^; ^1^H NMR (CDCl_3_): *δ* = 7.15 (t, *J* = 5.65 Hz, 1H, H-*p*, Phe), 7.05–6.97 (m, 2H, H-*m*, Phe), 6.76 (dd, *J* = 5.8 Hz, 7.15 Hz, 2H, H-*o*, Phe), 5.16 (t, *J* = 5.65 Hz, 1H, H-α, Phe), 4.11 (t, *J* = 6.45 Hz, 1H, H-α, Pro), 3.54 (s, 3H, *O*CH_3_), 3.21 (t, *J* = 7.15 Hz, 2H, H-*δ*, Pro), 3.09 (d, *J* = 4.85 Hz, 2H, H-*β*, Phe), 3.05 (s, 3H, *N*CH_3_), 2.57 (q, *J* = 5.45 Hz, 2H, H-*β*, Pro), 1.92-1.86 (m, 2H, H-*γ*, Pro), 1.46 (s, 9H, *tert*-Butyl); ^13^C NMR (CDCl_3_): *δ* = 175.4, 170.1 (2C, C=O, Pro and Phe), 159.7 (C=O, Boc), 138.8 (C-*γ*, Phe), 130.2 (2C, C-*o*, Phe), 127.6 (2C, C-*m*, Phe), 126.2 (C-*p*, Phe), 80.3 (C-*α*, *tert*-Butyl), 62.7, 59.3 (2C, C-*α*, Pro and Phe), 53.8 (*O*CH_3_), 46.9 (C-*δ*, Pro), 34.9, 32.7 (2C, C-*β*, Phe and Pro), 32.1 (*N*CH_3_), 29.5 (3C, C-*β*, *tert*-Butyl), 24.0 (C-*γ*, Pro); C_21_H_30_N_2_O_5_ (390): calcd. C 64.60, H 7.74, N 7.17; found C 64.59, H 7.71, N 7.19. 

### 4.2. Procedure for the Synthesis of Linear Tetrapeptide Segments (***3***)

To the solution of the dipeptide methyl ester, l-Pro-l-*N*-(Me)Phe-OMe (2.90 g, 0.01 mol) in tetrahydrofuran (THF, 25 mL), NMM (2.23 mL, 0.021 mol) was added at 0 °C, and the reaction mixture was stirred for 25 min. To a solution of Boc-l-Pro-l-*N*-(Me)Leu-OH (3.42 g, 0.01 mol) in THF (25 mL), DCC (2.12 g, 0.01 mol) and HOBt (1.34 g, 0.01 mol) were added with stirring and the resulting mixture was added to the above reaction mixture. The stirring of the resulting mixture was continued for 24 h at RT. This reaction was repeated using EDC·HCl (1.92 g, 0.01 mol) as a coupling agent in place of DCC. The reaction mixture was filtered and the residue was washed with THF (25 mL) and added to the filtrate. The filtrate was washed with 5% NaHCO_3_ and saturated NaCl solutions. The organic layer was dried over anhydrous Na_2_SO_4_, filtered, and evaporated in vacuum. The crude product was recrystallized from a mixture of chloroform and petroleum ether (b.p. 40–60 °C) followed by cooling at 0 °C to obtain the title compound Boc-l-Pro-l-*N*-(Me) Leu-l-Pro-l-*N*-(Me)Phe-OMe (**3**). 

#### *tert*-Butyloxycarbonyl-l-Prolyl-l-*N*-Methyl-l-Leucyl-l-Prolyl-l-*N*-Methyl-Phenylalanine Methyl Ester (**3**)

Semisolid mass; yield 83%; [*α*]_D_ = –53.6° (*c* = 0.25, MeOH); R*_f_* = 0.82 (CHCl_3_·MeOH-7:3); IR (CHCl_3_): *v* 2997-2986 (C–Hstr), 2962, 2925–2921, 2875 (C–Hstr), 2801–2794 (C–Hstr *N*-Me), 1742 (C=Ostr), 1676, 1645–1640 (C=O_str_), 1544, 1418 (C=C), 1395, 1373 (C–Hdef), 1386, 1362 (C–Hdef), 1273 (C–Ostr), 729, 684 (C–Hdef) cm^−1^; ^1^H NMR (CDCl_3_): *δ* = 7.19 (t, *J* = 5.65 Hz, 1H, H-*p*, Phe), 7.02–6.95 (m, 2H, H-*m*, Phe), 6.72 (dd, *J* = 5.75 Hz, 7.1 Hz, 2H, H-*o*, Phe), 5.38 (t, *J* = 5.55 Hz, 1H, H-α, Phe), 4.45 (t, *J* = 7.15 Hz, 1H, H-α, Pro-2), 4.36 (t, *J* = 6.15 Hz, 1H, H-α, Leu), 3.95 (t, *J* = 7.15 Hz, 1H, H-α, Pro-1), 3.68 (t, *J* = 7.2 Hz, 2H, H-*δ*, Pro-2), 3.54 (s, 3H, *O*CH_3_), 3.24 (t, *J* = 7.15 Hz, 2H, H-*δ*, Pro-1), 3.09 (d, *J* = 4.75 Hz, 2H, H-*β*, Phe), 3.06 (s, 3H, *N*CH_3_), 2.98 (s, 3H, *N*CH_3_), 2.71 (q, *J* = 5.45 Hz, 2H, H-*β*, Pro-2), 2.59 (q, *J* = 5.7 Hz, 2H, H-*β*, Pro-1), 1.98–1.89 (m, 4H, H-*γ*, Pro-2 and Pro-1), 1.94–1.83 (m, 3H, H-*β* and H-*γ*, Leu), 1.52 (s, 9H, *tert*-Butyl), 0.99 (d, *J* = 6.35 Hz, 6H, H-*δ*, Leu); ^13^C NMR (CDCl_3_): *δ* = 175.2, 169.3 (2C, C=O, Pro-2 and Leu), 167.8, 164.4 (2C, C=O, Phe and Pro-1), 155.4 (C=O, Boc), 139.4 (C-*γ*, Phe), 129.8 (2C, C-*o*, Phe), 127.9 (2C, C-*m*, Phe), 126.1 (C-*p*, Phe), 80.4 (C-*α*, *tert*-Butyl), 59.2 (C-*α*, Leu), 57.5 (C-*α*, Pro-2), 55.7(C-*α*, Pro-1), 53.9 (C-*α*, Phe), 53.0 (*O*CH_3_), 46.2, 43.6 (2C, C-*δ*, Pro-1 and Pro-2), 36.6 (C-*β*, Leu), 33.9 (*N*CH_3_), 33.0, 32.3 (2C, C-*β*, Phe and Pro-1), 31.7 (*N*CH_3_), 30.8 (C-*β*, Pro-2), 29.6 (3C, C-*β*, *tert*-Butyl), 27.2 (C-*γ*, Pro-1), 25.5 (2C, C-*δ*, Leu), 23.9 (C-*γ*, Pro-2), 22.2 (C-*γ*, Leu); C_33_H_50_N_4_O_7_ (614): calcd. C 64.47, H 8.20, N 9.11; found C 64.45, H 8.19, N 9.14. 

### 4.3. Procedure for the Synthesis of N-Methylated Tetracyclopeptide (***4***)

The linear tetrapeptide unit, Boc-l-Pro-l-*N*-(Me)Leu-l-Pro-l-*N*-(Me)Phe-OMe (**3**, 3.07 g, 0.005 mol) was deprotected at the carboxyl terminal using LiOH (0.18 g, 0.0075 mol) to obtain Boc-l-Pro-l-*N*-(Me)Leu-l-Pro-l-*N*-(Me)Phe-OH (**3a**). To a solution of the deprotected tetrapeptide **3a** (3.00 g, 0.005 mol) in CHCl_3_ (50 mL), *p*-nitrophenol (*pnp*, 0.94 g, 0.0067 mol) and *N*,*N*′-Diisopropylcarbodiimide (DIPC) (0.63 g, 0.005 mol) were added followed by stirring at room temperature for 12 h. The filtrate of the above reaction mixture was washed with 10% NaHCO_3_ (3 × 25 mL) and 5% HCl (2 × 25 mL) solutions to obtain the corresponding *p*-nitrophenyl ester. This reaction was repeated using pentafluorophenol (*pfp*, 1.23 g, 0.0067 mol) in place of *p*-nitrophenol, producing pentafluorophenyl ester. The Boc-group of the resulting units Boc-l-Pro-l-*N*-(Me)Leu-l-Pro-l-*N*-(Me)Phe-O*pnp* (2.89 g, 0.004 mol)/Boc-l-Pro-l-*N*-(Me)Leu-l-Pro-l-*N*-(Me)Phe-O*pfp* (3.07 g, 0.004 mol) was removed using TFA (0.91 g, 0.008 mol) to obtain the deprotected products l-Pro-l-*N*-(Me)Leu-l-Pro-l-*N*-(Me)Phe-O*pnp*/l-Pro-l-*N*-(Me)Leu-l-Pro-l-*N*-(Me)Phe-O*pfp,* which were dissolved in CHCl_3_ (20 mL), followed by the addition of TEA/NMM/C_5_H_5_N (2.8 mL/2.21 mL/1.61 mL, 0.021 mol). Then, all contents were maintained at 0 °C for 7 days. The reaction mixtures were washed with 10% NaHCO_3_ (3 × 20 mL) and 5% HCl (2 × 20 mL) solutions. The organic layer was dried over anhydrous Na_2_SO_4_ and _the_ crude cyclized compound was recrystallized using DCM and *n*-hexane to obtain the pure cyclic product *cyclo* (l-Pro-l-*N*-(Me)Leu-l-Pro-l-*N*-(Me)Phe) (**4**).

#### *Cyclo* (l-Prolyl-l-*N*-Methyl-l-Leucyl-l-Prolyl-l-*N*-Methyl-Phenylalanyl) (**4**)

Pale white solid; m.p. 189–190 °C (d); yield 89% (NMM), 78% (pyridine), 74% (TEA); [*α*]_D_ = −67.4° (*c* = 0.1, MeOH); R*_f_* = 0.59 (CHCl_3_·MeOH − 9:1); IR (KBr): *v* = 2999–2989 (C–Hstr), 2969, 2925, 2919, 2879 (C–Hstr), 2805-2797 (C–Hstr *N*-Me), 1674, 1643, 1636-1632 (C=Ostr), 1545, 1411 (C=C), 1382, 1366 (C–Hdef), 726, 688 (C–Hdef) cm^−1^; ^1^H NMR (CDCl_3_): *δ* = 7.38, 7.27 (dd, *J*=5.55 Hz, 7.15 Hz, 2H, H-*m*, Phe), 7.02 (t, *J* = 5.6 Hz, 1H, H-*p*, Phe), 6.73, 6.64 (dd, *J* = 5.8 Hz, 7.15 Hz, 2H, H-*o*, Phe), 5.47 (t, *J* = 6.1 Hz, 1H, H-α, Leu), 4.97 (t, *J* = 5.6 Hz, 1H, H-α, Phe), 4.48 (t, *J* = 7.15 Hz, 1H, H-α, Pro-2), 4.37 (t, *J* = 7.2 Hz, 1H, H-α, Pro-1), 3.63 (t, *J* = 7.2 Hz, 2H, H-*δ*, Pro-1), 3.48 (t, *J* = 7.15 Hz, 2H, H-*δ*, Pro-2), 3.02 (s, 3H, *N*CH_3_), 2.68 (s, 3H, *N*CH_3_), 2.41 (q, *J* = 5.5 Hz, 2H, H-*β*, Pro-2), 2.33 (q, *J* = 5.65 Hz, 2H, H-*β*, Pro-1), 2.14 (d, *J* = 4.7 Hz, 2H, H-*β*, Phe), 1.88–1.62 (m, 4H, H-*γ*, Pro-2 and Pro-1), 1.39 (t, *J* = 6.25 Hz, 2H, H-*β*, Leu), 0.97 (d, *J* = 6.3 Hz, 6H, H-*δ*, Leu), 0.77–0.62 (m, 1H, H-*γ*, Leu); ^13^C NMR (CDCl_3_): *δ* = 173.5, 171.2 (2C, C=O, Leu and Pro-2), 170.4, 168.8 (2C, C=O, Phe and Pro-1), 137.2 (C-*γ*, Phe), 130.6 (2C, C-*m*, Phe), 127.9 (2C, C-*o*, Phe), 126.5 (C-*p*, Phe), 58.8 (C-*α*, Phe), 55.5 (C-*α*, Pro-2), 54.2 (C-*α*, Pro-1), 51.8 (C-*α*, Leu), 48.3, 47.1 (2C, C-*δ*, Pro-1 and Pro-2), 39.2 (C-*β*, Phe), 37.4 (*N*CH_3_), 35.7 (*N*CH_3_), 34.1, 32.4 (2C, C-*β*, Leu and Pro-1), 31.5 (C-*β*, Pro-2), 28.5 (C-*γ*, Leu), 24.6 (2C, C-*δ*, Leu), 22.9 (C-*γ*, Pro-1), 21.8 (C-*γ*, Pro-2); FABMS: *m*/*z* = 483 [(M + H)^+^, 100], 455 [(483–CO)^+^, 15], 386 [(H*N*(Me)Leu-Pro-*N*(Me)Phe)^+^, 56], 358 [(386–CO)^+^, 22], 356 [Pro-*N*(Me)Phe-Pro)^+^, 42], 328 [(356–CO)^+^, 18], 322 [(Pro-*N*(Me)Leu-Pro)^+^, 39], 294 [(322–CO)^+^, 19], 259 [(H*N*(Me)Phe-Pro)^+^, 66], 231 [(259–CO)^+^, 19], 225 [(H*N*(Me)Leu-Pro)^+^, 47], 197 [(225–CO)^+^, 27], 162 [(H*N*(Me)Phe)^+^, 29], 134 [*N*(Me)Phe immonium ion (C_9_H_12_N)^+^, 16], 128 [(H*N*(Me)Leu)^+^, 32], 100 [*N*(Me)Leu immonium ion (C_6_H_14_N)^+^, 14], 98 [(Pro)^+^, 28], 91 [(C_7_H_7_)^+^, 9], 77 [(C_6_H_5_)^+^, 10], 70 [Pro immonium ion (C_4_H_8_N)^+^, 21]; 57 [(C_4_H_9_)^+^, 8], 43 [(C_3_H_7_)^+^, 11], 15 [(CH_3_)^+^, 19]; C_27_H_38_N_4_O_4_ (482): calcd. C 67.20, H 7.94, N 11.61; found C 67.19, H 7.95, N 11.63. 

### 4.4. Pharmacological Studies 

#### 4.4.1. Anthelmintic Screening

The anthelmintic activity studies for the newly synthesized linear and cyclic *N*-methylated tetrapeptides (**3**, **4**) were carried out against the three different species of the earthworms *M. konkanensis*, *P. corethruses*, and *E. eugeniae* at a concentration of 2 mg/mL. Suspensions of the samples were prepared by triturating the synthesized compounds (100 mg) with Tween 80 (0.5%) and distilled water and the resulting mixtures were stirred using a mechanical stirrer for 30 min. The suspensions were diluted to contain 0.2% *w/v* of the test samples. Suspensions of the reference drug, Mebendazole, were prepared with the same concentration in a similar way. Five earthworms per earthworm species under investigation were evaluated in triplicate. Earthworms of similar size (2 inches in length) were placed in Petri plates having a 4-inch diameter, containing 50 mL of the suspension of the test sample and the reference drug at room temperature (RT). Another set of five earthworms was maintained as a control in 50 mL of suspension of the distilled water and Tween 80 (0.5%). The paralysis and death times were noted and their mean was calculated for the triplicate sets. The death time was ascertained by placing the earthworms in warm water (50 °C), which stimulated movement if the worm was alive. The results of the anthelmintic studies are compiled in Table 1.

#### 4.4.2. Antibacterial Screening

The newly synthesized linear and cyclic *N*-methylated proline-rich tetrapeptides (**3**, **4**) were evaluated for their antibacterial potential against two Gram-positive bacteria (*B. subtilis* and *S. aureus*) and two Gram-negative bacteria (*P. aeruginosa* and *K. pneumoniae*) at concentrations ranging from 6.25–50 μg/mL. The minimum inhibitory concentration (MIC) values of test compounds were determined by the Tube Dilution Technique. Both the linear and cyclic tetrapeptides were dissolved separately to prepare a stock solution of 1 mg/mL using dimethylformamide (DMF). The stock solution was aseptically transferred and suitably diluted with the sterile broth medium to contain seven different concentrations of each test compound, ranging from 3.1–200 μg/mL in different test tubes. The tubes were inoculated with one loopful of overnight growth culture of the test bacteria. The process was repeated with the different test bacteria and the different samples. The tubes inoculated with bacterial cultures were incubated at 37 °C for 18 h and the presence/absence of growth of the bacteria was observed. From these results, the MIC of each test compound was determined against each test bacterium. A possible spore suspension was prepared in sterile distilled water from 5-day-old culture of the test bacteria growing on nutrient broth media. About 20 mL of the growth medium was transferred into the sterilized Petri plates and inoculated with 1.5 mL of the spore suspension (spore concentration ~ 6 × 10^4^ spores/mL). Filter paper disks having a 6-mm diameter and a 1-mm thickness were sterilized by autoclaving at 121 °C (15 psi) for 15 min. Each Petri plate was divided into five equal portions along the diameter to place one disc. Three discs of the test sample were placed on three portions together with one disc with the reference drug (Gatifloxacin) and a disk impregnated with the solvent (DMF) as the negative control. The Petri plates inoculated with bacterial cultures were incubated at 37 °C for 18 h. Diameters of the zones of inhibition (ZOI in mm) were measured and the average diameters of the test samples were calculated in triplicate. The diameters obtained for the test samples were compared with that produced by the standard drug. The results of the antibacterial studies are presented in Table 2.

#### 4.4.3. Antifungal Screening

The serial plate dilution method was used for the evaluation of antifungal activity against the diamorphic fungal strain *C. albicans* and three other fungal strains, including *A. niger* and two cutaneous fungal strains, *M. audouinii* and *T. mentagrophytes*, at concentrations ranging from 6.25–50 μg/mL for the newly synthesized linear and cyclic tetrapeptides (**3**, **4**). The MIC values of the test compounds were determined by employing the same technique as that used for the antibacterial studies using dimethyl sulfoxide (DMSO) instead of DMF, and the tubes inoculated with fungal cultures were incubated at 37 °C for 48 h. After incubation, the presence/absence of the fungal growth was observed and MIC of the test compounds was determined against each test fungus. A spore suspension in normal saline (0.91% *w*/*v* of NaCl) was prepared from the culture of the test fungi on Sabouraud’s broth media. After transferring the growth medium, the Petri plates were inoculated with the spore suspension. After drying, wells were made using an agar punch and the test samples, reference drug (Griseofulvin), and negative control (DMSO) were placed in the labeled wells in each Petri plate. The Petri plates inoculated with the fungal cultures were incubated at 37 °C for 48 h. Antifungal activity was determined by measuring the diameter of the inhibition zone for the triplicate sets. The activity of each compound was compared with the reference standard. The results of the antifungal studies are given in Table 2.

The experimental details of the biological activity studies are described in our earlier reports [43,44,45,46,47,48,49,50,51,52]. Further, in order to describe the intermolecular forces of drug receptor interaction as well as the transport and distribution of drugs in a quantitative manner, various steric and the lipophilicity parameters need to be calculated for the synthesized linear and cyclic tetracyclopeptides (**3**, **4**). As per International Union of Pure and Applied Chemistry (IUPAC) rules, tetracyclopeptide **4** can be named as 6-Benzyl-14-isobutyl-7,15-dimethylperhydrodipyrrolo[1,2-a:1,2-g] [1,4,7,10] tetraazacyclododecine-5,8,13,16-tetraone.

## 5. Conclusions

An efficient strategy was developed toward the first total synthesis of an *N*-methylated analog of tetracyclopeptide (**4**) utilizing carbodiimide chemistry. The EDC·HCl/NMM coupling method provided 9–10% additional yield in comparison to the methods utilizing EDC·HCl/TEA and DCC/TEA or NMM. The *p*-nitrophenyl ester was shown to be better for the activation of the acid functionality of the linear tetrapeptide unit in comparison to pentafluorophenyl ester. The NMM was found to be a better base for the intramolecular cyclization of the linear peptide segment in comparison to pyridine. Like other proline-containing synthetic cyclooligopeptides, the synthesized *N*-methylated tetracyclopeptide displayed potent anthelmintic activity against *M. konkanensis*, *P. corethruses*, and *E. eugeniae* as well as effectiveness against pathogenic *M. audouinii*, *T. mentagrophytes*, and *C. albicans*. Further, Gram-negative bacteria were found to be more sensitive than Gram-positive bacteria towards the newly synthesized tetracyclopeptide at the concentrations tested. The improved pharmacological activity results of the *N*-methylated analog further proved the potential of *N*-methylation in modulating biological activity and selectivity. On passing toxicity tests, tetracyclopeptide **4** may prove to be a good candidate for clinical studies and perhaps eventual consideration as a new anthelmintic and antifungal drug.

## Figures and Tables

**Table 1:** Anthelmintic activity data for the proline-rich linear and cyclic tetracyclopeptides **3**, **4**.

Compound *^Ә^*	Earthworm Species					
	*M. kon.*		*P. cor.*		*E. eug.*	
	Mean Paralyzing Time (min) ^‡^	Mean Death Time (min) ^‡^	Mean Paralyzing Time (min)	Mean Death Time (min)	Mean Paralyzing Time (min)	Mean Death Time (min)
III *	14.06 ± 0.36	22.09 ± 0.27	18.55 ± 0.19	29.44 ± 0.29	14.15 ± 0.26	24.32 ± 0.36
IV *	10.25 ± 0.22	18.22 ± 0.14	12.36 ± 0.37	21.25 ± 0.14	12.34 ± 0.41	21.04 ± 0.21
**3**	13.43 ± 0.22	21.13 ± 0.49	17.08 ± 0.16	27.16 ± 0.22	12.57 ± 0.33	22.08 ± 0.25
**4**	08.12 ± 0.41	16.45 ± 0.26	09.55 ± 0.29	20.05 ± 0.44	09.50 ± 0.24	18.05 ± 0.39
Control ^#^	-	-	-	-	-	-
Mebendazole	13.58 ± 0.38	22.59 ± 0.29	17.58 ± 0.40	29.56 ± 0.15	13.50 ± 0.44	24.09 ± 0.49

*M. kon.*: *Megascoplex konkanensis*; *P. cor.*: *Pontoscotex corethruses; E. eug.*: *Eudrilus eugeniae*. **^‡^** Data are given as mean ± S.D. (*n* = 3). * Bioactivity data for III, IV (non-methylated compounds) were obtained under the same experimental conditions as those used for methylated derivatives and compared with the results of a previously published report [27]. ^#^ Tween 80 (0.5%) in distilled water. *^Ә^* Compounds were tested at a concentration of 2 mg/mL.

**Table 2:** Antimicrobial activity data for proline-rich linear and cyclic tetracyclopeptides **3**, **4**.

Compound *^Ә^*	Diameter of Zone of Inhibition (mm)							
	Bacterial Strains				Fungal Strains			
	*B. sub.*	*S. aur.*	*P. aer.*	*K. pne.*	*C. alb.*	*M. aud.*	*A. nig.*	*T. men.*
III *	-	-	12 (25) ^†^	17 (25)	9 (12.5)	17 (6)	-	18 (6)
IV *	-	-	16 (25)	19 (25)	13 (12.5)	25 (6)	-	26 (6)
**3**	-	10 (12.5)	16 (12.5)	18 (12.5)	18 (6)	19 (6)	-	22 (6)
**4**	-	12 (12.5)	21 (12.5)	23 (12.5)	24 (6)	27 (6)	-	28 (6)
Control ^#^	-	-	-	-	-	-	-	-
Gatifloxacin	18 (12.5) ^†^	27 (6)	23(6)	26 (6)	-	-	-	-
Griseofulvin	-	-	-	-	20 (6)	18 (6)	20 (12.5)	19 (6)

*B. sub.*: *Bacillus subtilis*; *S. aur.*: *Staphylococcus aureus*; *P. aer.*: *Pseudomonas aeruginosa*; *K. pne.*: *Klebsiella pneumoniae*; *C. alb.*: *Candida albicans*; *M. aud.*: *Microsporum audouinii*; *A. nig.*: *Aspergillus niger*; *T. men.*: *Trichophyton mentagrophytes*. * Bioactivity data for III, IV (non-methylated compounds) were obtained under the same experimental conditions as those used for methylated derivatives and compared with the results of a previously published report [27]. ^†^ Values in brackets are minimum inhibitory concentration (MIC) values (μg/mL). ^#^ Dimethylformamide/Dimethyl sulfoxide (DMF/DMSO), *^Ә^* Compounds were tested at a concentration in the range of 6–25 μg/mL.

## Supplementary Materials

The following are available online at http://www.mdpi.com/1660-3397/16/9/305/s1, Figures S1–S3: ^1^H NMR, ^13^C NMR and mass spectrum for newly synthesized *N*-methylated proline-rich tetracyclopeptide, Table S1: Various steric and the lipophilicity parameters for the linear and cyclic tetracyclopeptides.
