# Supplementary material for: Toward the Synthesis and Improved Biopotential of an N-methylated Analog of a Proline-Rich Cyclic Tetrapeptide from Marine Bacteria
Source: Mar Drugs. 2018 Aug 30;16(9):305. doi: 10.3390/md16090305 (PMC6163976; doi:10.3390/md16090305)
Supplement: Supplementary file 1 [file marinedrugs-16-00305-s001.zip › marinedrugs-325713-SI.pdf]

Supporting Information

Toward the Synthesis and Improved Biopotential of an *N*-methylated Analog of a Proline-Rich Cyclic Tetrapeptide from Marine Bacteria

Rajiv Dahiya[1]*, Suresh Kumar[2]*, Sukhbir Lal Khokra[2] , Sheeba Varghese Gupta[3], Vijaykumar B. Sutariya[3], Deepak Bhatia[4], Ajay Sharma[5], Shamjeet Singh[1] and Sandeep Maharaj[1]

[1]Laboratory of Peptide Research and Development, School of Pharmacy, Faculty of Medical Sciences, The University of the West Indies, St. Augustine, Trinidad & Tobago, West Indies; Rajiv.Dahiya@sta.uwi.edu; Shamjeet.Singh@sta.uwi.edu; Sandeep.Maharaj@sta.uwi.edu
[2]Institute of Pharmaceutical Sciences, Kurukshetra University, Kurukshetra 136119, Haryana, India; sureshmpharma@rediffmail.com; slkhokra@kuk.ac.in
[3]Department of Pharmaceutical Sciences, USF College of Pharmacy, University of South Florida, Tampa, FL 33612-4749, USA; svarghes@health.usf.edu; vsutariy@health.usf.edu
[4]Department of Pharmacogenomics, Bernard J. Dunn School of Pharmacy, Shenandoah University- ICPH Fairfax, Fairfax, VA 22031, USA; dbhatia@su.edu
[5]Department of Pharmacognosy, Amity Institute of Pharmacy, Amity University, Gwalior 474020, Madhya Pradesh, India; asharma5@gwa.amity.edu

Content *Page*

Figure S1.
^{1}H NMR spectrum for proline-rich *N*-methylated tetracyclopeptide……………………….2

Figure S2.
^{13}C NMR spectrum for proline-rich *N*-methylated tetracyclopeptide……………………..3

Figure S3.
Mass spectrum for proline-rich *N*-methylated tetracyclopeptide……………………….4

Table S1.
Various steric and lipophilicity parameters for linear and tetracyclopeptide................5

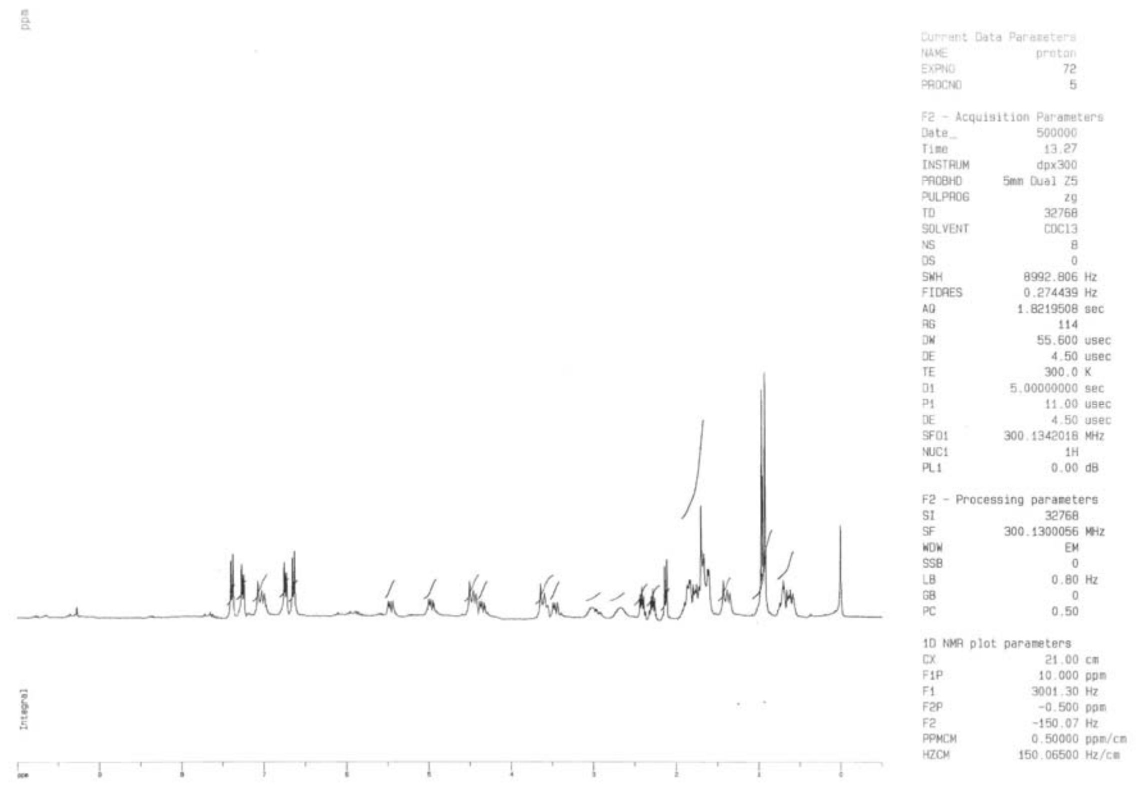

Figure S1. ^{1}H NMR spectrum for proline-rich N-methylated tetracyclopeptide **4**

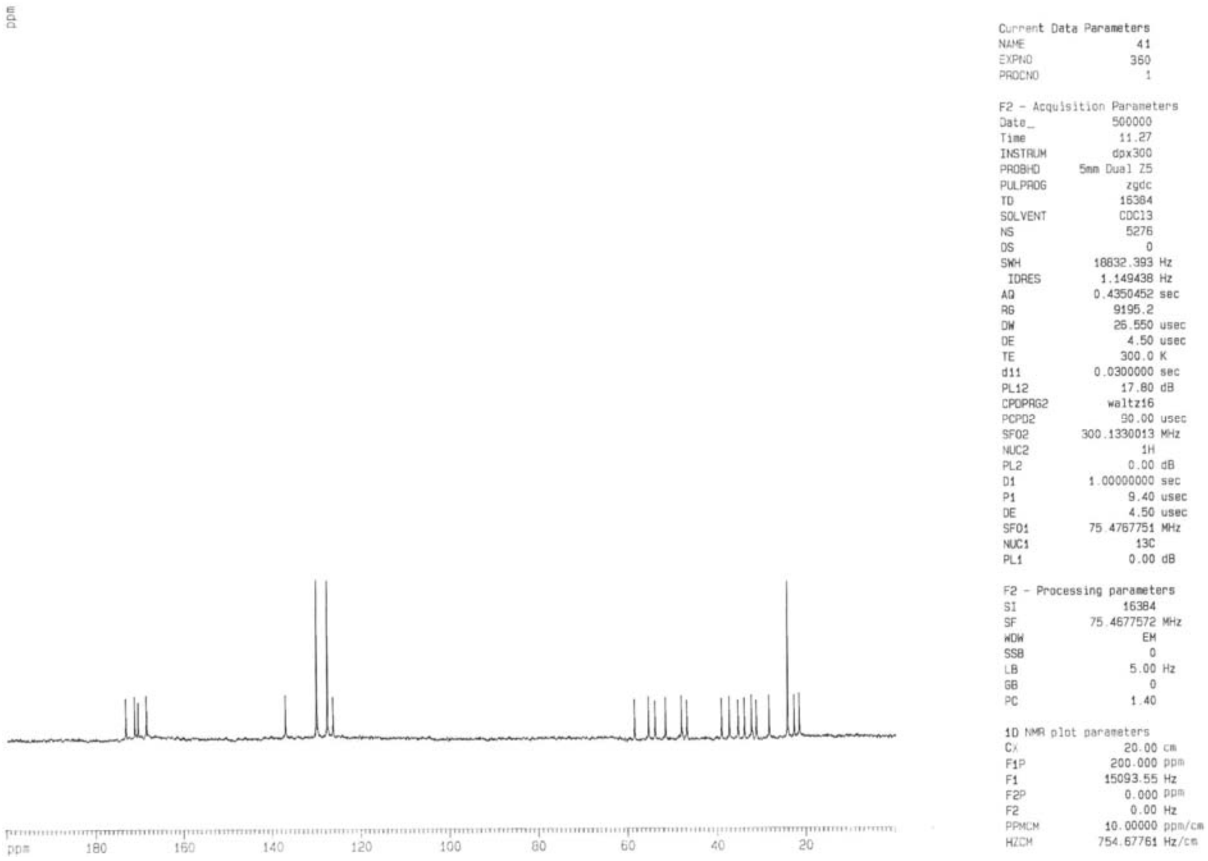

Figure S2. ^{13}C NMR spectrum for proline-rich N-methylated tetracyclopeptide **4**

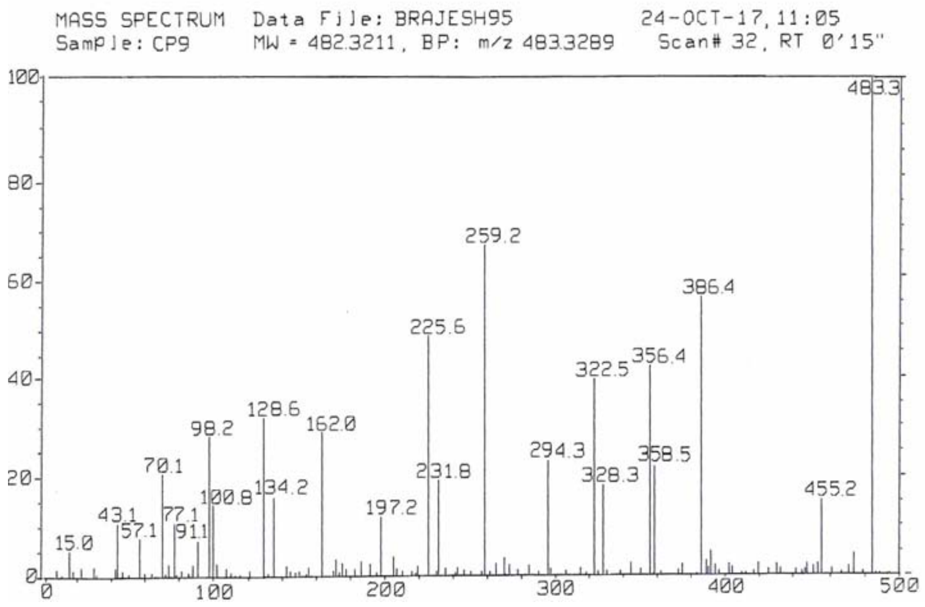

Figure S3. Mass spectrum for proline-rich *N*-methylated tetracyclopeptide **4**

Table S1. Various steric and lipophilicity parameters for linear and cyclotetrapeptide (**3**, **4**)

Parameter	*Calculated value for	
	**Compound 3**	**Compound 4**
Molar Refractivity (MR^{20})	165.05 ± 0.3 cm^3	133.74 ± 0.4 cm^3
Molar Volume (MV^{20})	523.8 ± 3.0 cm^3	391.9 ± 5.0 cm^3
Parachor (P_r)	1384.4 ± 6.0 cm^3	1072.4 ± 6.0 cm^3
Refractive Index (n^{20})	1.542 ± 0.02	1.598 ± 0.03
Surface Tension (γ^{20})	48.7 ± 3.0 dyne/cm	56.0 ± 5.0 dyne/cm
Density (d^{20})	1.173 ± 0.06 g cm^{-3}	1.23 ± 0.1 g cm^{-3}
Polarizability (α)	65.43 ± 0.5 10^{-24} cm^3	53.02 ± 0.5 10^{-24} cm^3
log P (n-Octanol/water)	4.19 ± 0.78	3.86 ± 0.89

*Values were calculated using ACD/ChemSketch 2.0 software
